# Supplementary material for: A global survey of arsenic-related genes in soil microbiomes
Source: BMC Biol. 2019 May 30;17:45. doi: 10.1186/s12915-019-0661-5 (PMC6543643; doi:10.1186/s12915-019-0661-5)
Supplement: Supplementary file 1 — Available metadata and accession numbers for soil metagenomes used in this study. (DOCX 22 kb) [file 12915_2019_661_MOESM1_ESM.docx]

| Project Name | Sample Location | Country | Sample Shortname | Accession location | Project ID | Sample Name | Gbp |
| --- | --- | --- | --- | --- | --- | --- | --- |
| ARMO | Rondonia | Brazil | Brazilian_forest | MG-RAST | mgp3731 | mgm4546395.3 | 13.27 |
| ARMO | Rondonia | Brazil | Brazilian_forest | MG-RAST | mgp3731 | mgm4536139.3 | 9.04 |
| ARMO | Rondonia | Brazil | Brazilian_forest | MG-RAST | mgp3731 | mgm4535554.3 | 9.69 |
| Axel Heiberg Permafrost: Part 4A | Central Axel Heiberg Island | Canada | Permafrost_Canada | MG-RAST | mgp252 | mgm4523023.3 | 6.52 |
| Axel Heiberg Permafrost: Part 4A | Central Axel Heiberg Island | Canada | Permafrost_Canada | MG-RAST | mgp252 | mgm4523145.3 | 5.52 |
| CedarCreek_minsoil_June2013 | Bethel, MN | USA | Minnesota_creek | MG-RAST | mgp5588 | mgm4541646.3 | 10.65 |
| CedarCreek_minsoil_June2013 | Bethel, MN | USA | Minnesota_creek | MG-RAST | mgp5588 | mgm4541645.3 | 9.77 |
| Fermi-syntheticlongreads | Fermi National Accelerator Laboratory | USA | Illinois_switchgrass | MG-RAST | mgp14596 | mgm4653791.3 | 7.95 |
| GED prairie unassembled | Iowa | USA | Iowa_prairie | MG-RAST | mgp6377 | mgm4539575.3 | 18.79 |
| GED prairie unassembled | Iowa | USA | Iowa_prairie | MG-RAST | mgp6377 | mgm4539572.3 | 17.58 |
| GED prairie unassembled | Iowa | USA | Iowa_prairie | MG-RAST | mgp6377 | mgm4539576.3 | 17.43 |
| GP corn unassembled | Iowa | USA | Iowa_corn | MG-RAST | mgp6368 | mgm4539522.3 | 8.19 |
| GP corn unassembled | Iowa | USA | Iowa_corn | MG-RAST | mgp6368 | mgm4539523.3 | 8.12 |
| Hofmockel Soil Aggregate COB KBASE | Boone County, IA | USA | Iowa_agricultural | MG-RAST | mgp2592 | mgm4509400.3 | 24.98 |
| Hofmockel Soil Aggregate COB KBASE | Boone County, IA | USA | Iowa_agricultural | MG-RAST | mgp2592 | mgm4509401.3 | 7.86 |
| ISA-SMC-2011 | Auburn, IL | USA | Illinois_soybean | MG-RAST | mgp2076 | mgm4502542.3 | 12.54 |
| ISA-SMC-2011 | Auburn, IL | USA | Illinois_soybean | MG-RAST | mgp2076 | mgm4502540.3 | 10.60 |
| Loma_Ridge_grassland | Loma Ridge, CA | USA | California_grassland | MG-RAST | mgp1992 | mgm4511115.3 | 6.50 |
| Loma_Ridge_grassland | Loma Ridge, CA | USA | California_grassland | MG-RAST | mgp1992 | mgm4511062.3 | 5.77 |
| Mining of new genes and pathways from soil of mangrove forest | Matang Mangrove Forest | Malaysia | Mangrove | MG-RAST | mgp11628 | mgm4603402.3 | 24.38 |
| Mining of new genes and pathways from soil of mangrove forest | Matang Mangrove Forest | Malaysia | Mangrove | MG-RAST | mgp11628 | mgm4603270.3 | 24.54 |
| NEON | Disney Wilderness Preserve, FL | USA | Disney_preserve | MG-RAST | mgp13948 | mgm4664918.3 | 11.20 |
| NEON | Disney Wilderness Preserve, FL | USA | Disney_preserve | MG-RAST | mgp13948 | mgm4664925.3 | 4.14 |
| Permafrost sediments, North-East Siberia, Kolyma lowland | Kolyma river lowland | Russia | Permafrost_Russia | MG-RAST | mgp7176 | mgm4546813.3 | 19.20 |
| Ungulate Exclosure 2015 | Wyoming | USA | Wyoming_soil | MG-RAST | mgp15600 | mgm4670120.3 | 6.41 |
| Surface soil microbial communities from Centralia Pennsylvania | Centralia, PA | USA | Centralia_recovered | JGI | Gp0112853 | Cen01 | 23 |
| Surface soil microbial communities from Centralia Pennsylvania | Centralia, PA | USA | Centralia_recovered | JGI | Gp0112853 | Cen03 | 26 |
| Surface soil microbial communities from Centralia Pennsylvania | Centralia, PA | USA | Centralia_recovered | JGI | Gp0112853 | Cen04 | 25 |
| Surface soil microbial communities from Centralia Pennsylvania | Centralia, PA | USA | Centralia_recovered | JGI | Gp0112853 | Cen05 | 25 |
| Surface soil microbial communities from Centralia Pennsylvania | Centralia, PA | USA | Centralia_fire-affected | JGI | Gp0112853 | Cen06 | 22 |
| Surface soil microbial communities from Centralia Pennsylvania | Centralia, PA | USA | Centralia_recovered | JGI | Gp0112853 | Cen07 | 21 |
| Surface soil microbial communities from Centralia Pennsylvania | Centralia, PA | USA | Centralia_fire-affected | JGI | Gp0112853 | Cen10 | 36 |
| Surface soil microbial communities from Centralia Pennsylvania | Centralia, PA | USA | Centralia_fire-affected | JGI | Gp0112853 | Cen12 | 24 |
| Surface soil microbial communities from Centralia Pennsylvania | Centralia, PA | USA | Centralia_fire-affected | JGI | Gp0112853 | Cen14 | 24 |
| Surface soil microbial communities from Centralia Pennsylvania | Centralia, PA | USA | Centralia_fire-affected | JGI | Gp0112853 | Cen15 | 20 |
| Surface soil microbial communities from Centralia Pennsylvania | Centralia, PA | USA | Centralia_fire-affected | JGI | Gp0112853 | Cen16 | 51 |
| Surface soil microbial communities from Centralia Pennsylvania | Centralia, PA | USA | Centralia_reference | JGI | Gp0112853 | Cen17 | 24 |
| Surface soil microbial communities from an active vent of coal mine fire in Centralia Pennsylvania, Sep 20 ‘18 | Centralia, PA | USA | Centralia_fire-affected | NCBI | SRR7882662 | Cen13 | 56 |
