# Supplementary material for: A global survey of arsenic-related genes in soil microbiomes
Source: BMC Biol. 2019 May 30;17:45. doi: 10.1186/s12915-019-0661-5 (PMC6543643; doi:10.1186/s12915-019-0661-5)
Supplement: Supplementary file 2 — Phylum-level summary of arsenic-related genes in RefSoil+ chromosomes and plasmids. (DOCX 14 kb) [file 12915_2019_661_MOESM2_ESM.docx]

| Phylum | Chromosome | | | | | | | | | Plasmid | | | | | | |
| --- | --- | --- | --- | --- | --- | --- | --- | --- | --- | --- | --- | --- | --- | --- | --- | --- |
|  | **arsB** | **acr3** | **arsC (grx)** | **arsC (trx)** | **arsM** | **aioA** | **arxA** | **arrA** | **arsB** | | **acr3** | **arsC (grx)** | **arsC (trx)** | **arsM** | **aioA** |  |
| Acidobacteria (7) | 57.1 | 100 | 0 | 14.3 | 14.3 | 0 | 0 | 0 | 0 | | 0 | 0 | 0 | 0 | 0 |  |
| Actinobacteria (118) | 44.1 | 61.9 | 33.1 | 5.9 | 1.7 | 0 | 0 | 0 | 0 | | 4.2 | 0 | 0.8 | 0.8 | 0 |  |
| Bacteroidetes (19) | 0 | 73.7 | 31.6 | 36.8 | 21.1 | 0 | 0 | 0 | 0 | | 5.3 | 0 | 0 | 0 | 0 |  |
| Chloroflexi (9) | 88.9 | 88.9 | 0 | 0 | 100 | 0 | 0 | 0 | 0 | | 0 | 0 | 0 | 0 | 0 |  |
| Cyanobacteria (26) | 3.8 | 76.9 | 11.5 | 34.6 | 3.8 | 0 | 0 | 0 | 0 | | 0 | 0 | 3.8 | 0 | 0 |  |
| Deinococcus-Thermus (6) | 50 | 0 | 0 | 16.7 | 0 | 0 | 0 | 0 | 16.7 | | 0 | 0 | 16.7 | 0 | 0 |  |
| Firmicutes (207) | 65.7 | 44.4 | 1 | 45.9 | 5.8 | 0 | 0 | 1.4 | 0 | | 3.4 | 0 | 1.9 | 0 | 0 |  |
| Proteobacteria (531) | 33.1 | 40.7 | 79.5 | 4 | 1.9 | 2.4 | 0.2 | 0.9 | 2.1 | | 4.1 | 5.6 | 0 | 0 | 0.4 |  |
| Spirochaetes (12) | 0 | 25 | 0 | 16.7 | 0 | 0 | 0 | 0 | 0 | | 0 | 0 | 0 | 0 | 0 |  |
