# Supplementary figures and images for: A global survey of arsenic-related genes in soil microbiomes
Source: BMC Biol. 2019 May 30;17:45. doi: 10.1186/s12915-019-0661-5 (PMC6543643; doi:10.1186/s12915-019-0661-5)

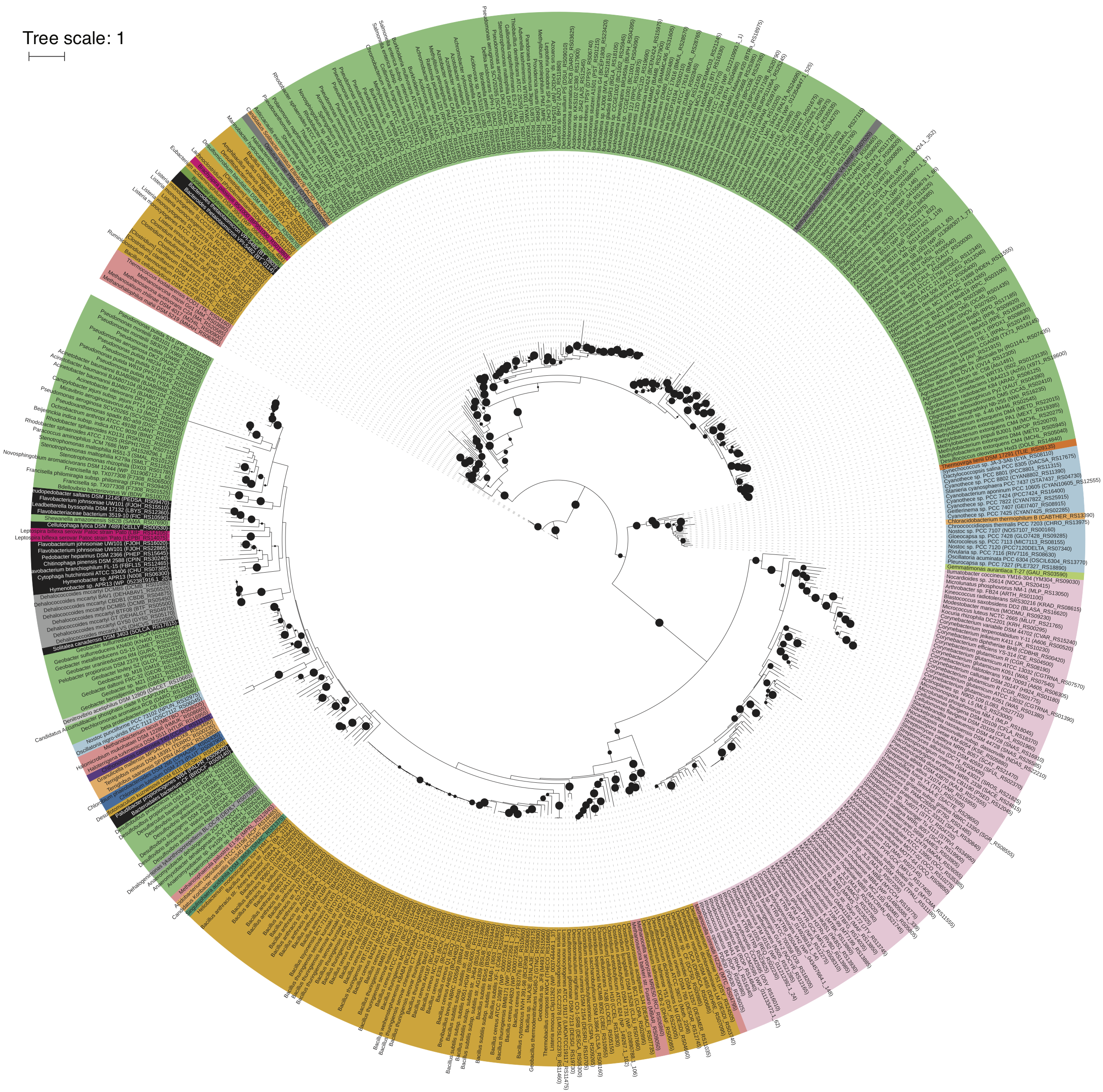

Supplement: Supplementary file 3 — Phylogeny of Acr3 in RefSoil+ organisms. Maximum likelihood tree with 100 bootstrap replications of Acr3 sequences predicted from RefSoil+ genomes. Leaf tips show the name of the RefSoil+ organisms and background color indicates phylum-level taxonomy. Bootstrap values > 50 are represented by black circles within the tree. (PNG 4395 kb) [file 12915_2019_661_MOESM3_ESM.png]

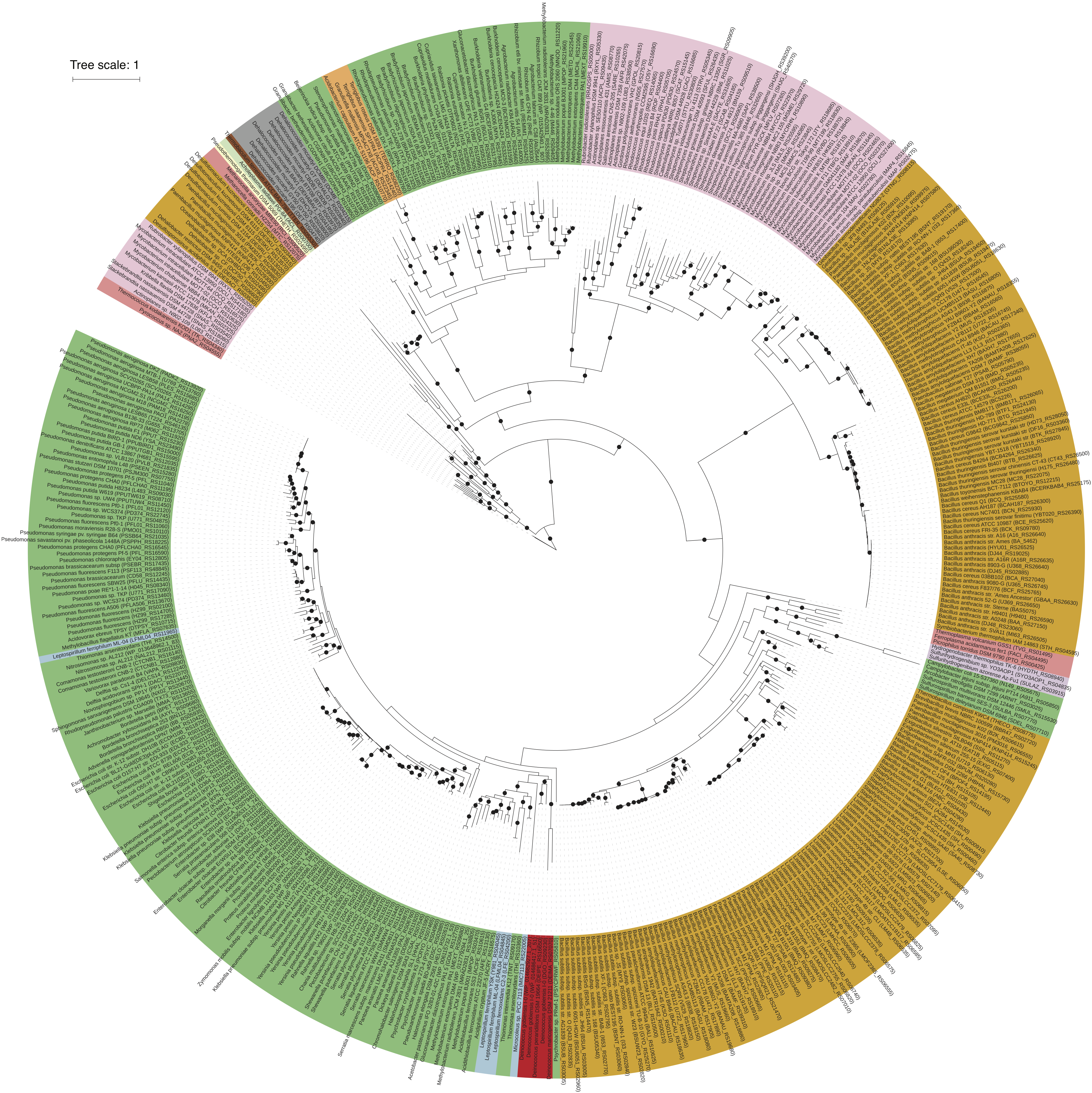

Supplement: Supplementary file 4 — Phylogeny of ArsB in RefSoil+ organisms. Maximum likelihood tree with 100 bootstrap replications of ArsB sequences predicted from RefSoil+ genomes. Leaf tips show the name of the RefSoil+ organisms and background color indicates phylum-level taxonomy. Bootstrap values > 50 are represented by black circles within the tree. (PNG 9385 kb) [file 12915_2019_661_MOESM4_ESM.png]

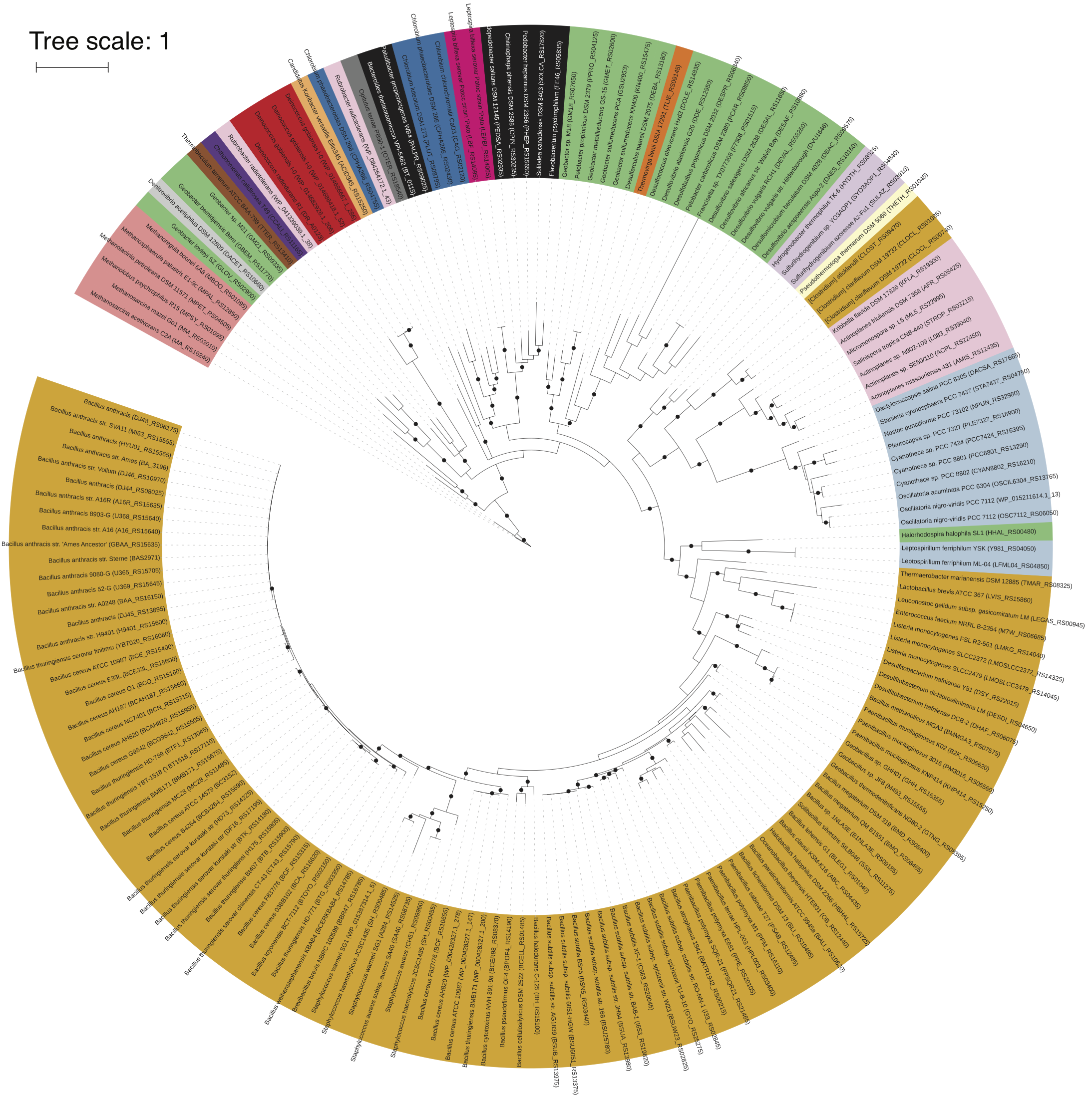

Supplement: Supplementary file 5 — Phylogeny of ArsC (trx) in RefSoil+ organisms. Maximum likelihood tree with 100 bootstrap replications of ArsC (trx) sequences predicted from RefSoil+ genomes. Leaf tips show the name of the RefSoil+ organisms and background color indicates phylum-level taxonomy. Bootstrap values > 50 are represented by black circles within the tree. (PNG 1911 kb) [file 12915_2019_661_MOESM5_ESM.png]

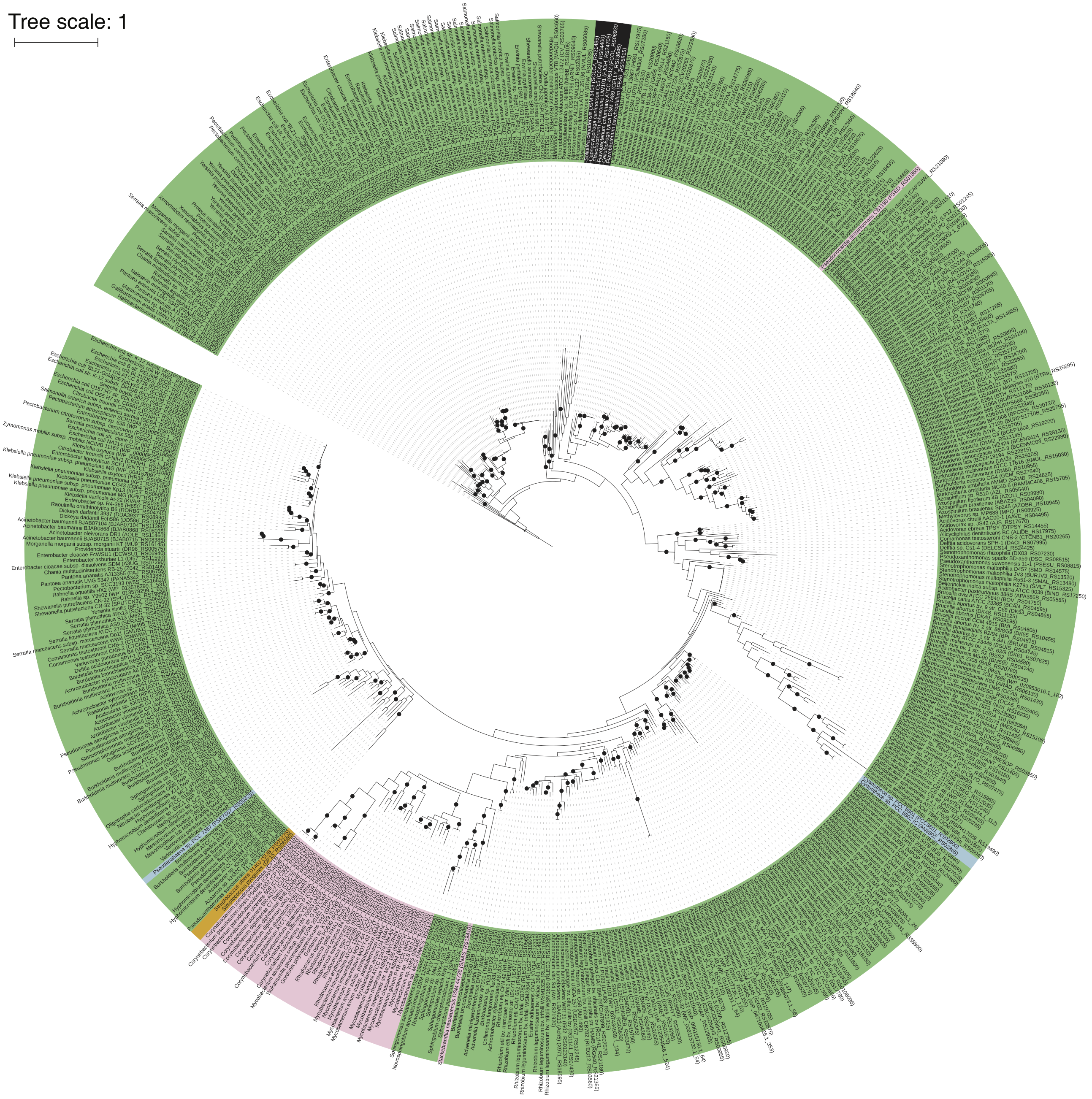

Supplement: Supplementary file 6 — Phylogeny of ArsC (grx) in RefSoil+ organisms. Maximum likelihood tree with 100 bootstrap replications of ArsC (grx) sequences predicted from RefSoil+ genomes. Leaf tips show the name of the RefSoil+ organisms and background color indicates phylum-level taxonomy. Bootstrap values > 50 are represented by black circles within the tree. (PNG 4752 kb) [file 12915_2019_661_MOESM6_ESM.png]
