# Supplementary material for: A global survey of arsenic-related genes in soil microbiomes
Source: BMC Biol. 2019 May 30;17:45. doi: 10.1186/s12915-019-0661-5 (PMC6543643; doi:10.1186/s12915-019-0661-5)
Supplement: Supplementary file 10 — Summary of endemic arsenic-related gene sequences. A sequence was considered endemic if it was present in less than three different soil sites. (DOCX 43 kb) [file 12915_2019_661_MOESM10_ESM.docx]

| **Gene** | **Number of sequences** | **Number of endemic sequences** | **Percent endemic** |
| --- | --- | --- | --- |
| *acr3* | 610 | 607 | 99.5 |
| *aioA* | 63 | 62 | 98.4 |
| *arrA* | 63 | 63 | 100 |
| *arsB* | 8 | 8 | 100 |
| *arsC* (grx) | 1316 | 1299 | 98.7 |
| *arsC* (trx) | 292 | 291 | 99.7 |
| *arsD* | 64 | 64 | 100 |
| *arsM* | 1193 | 1191 | 99.8 |
| *arxA* | 12 | 12 | 100 |
| **Totals** | 3621 | 3597 | 99.3 |
