# Supplementary material for: A global survey of arsenic-related genes in soil microbiomes
Source: BMC Biol. 2019 May 30;17:45. doi: 10.1186/s12915-019-0661-5 (PMC6543643; doi:10.1186/s12915-019-0661-5)
Supplement: Supplementary file 11 — Summary of reference arsenic resistance and metabolism gene sequences from FunGene databases. (DOCX 51 kb) [file 12915_2019_661_MOESM11_ESM.docx]

| FunGene Database/ Protein | Minimum HMM score | Minimum length (aa) | Minimum HMM coverage (%) | Number of FunGene sequences | Number of dereplicated sequences | Minimum assembled length (aa) |
| --- | --- | --- | --- | --- | --- | --- |
| ArsB | 150 | 400 | 80 | 23680 | 5250 | 150 |
| ACR3 | 140 | 300 | 80 | 19812 | 8002 | 150 |
| ArsC_glut | 80 | 120 | 85 | 18082 | 9635 | 50 |
| ArsC_thio | 172 | 100 | 80 | 7180 | 7180 | 50 |
| ArrA | 175 | 75 | 5 | 1621 | 1487 | 150 |
| AioA | 800 | 800 | 80 | 382 | 293 | 150 |
| ArsM | 200 | 100 | 30 | 3446 | 2948 | 160 |
| ArsD | 80 | 100 | 80 | 5404 | 876 | 150 |
| ArxA | 600 | 800 | 80 | 67 | 54 | 150 |
